# Effect of A Patient Reminder Program on Adherence in Postmenopausal Women with Osteoporosis Receiving Oral Bisphosphonate Treatment: A Randomized Clinical Control Trial

**DOI:** 10.1007/s00223-025-01405-6

**Published:** 2025-07-16

**Authors:** U. Stumpf, I. Kyvernitakis, K. Horas, P. Hadji

**Affiliations:** 1 Department of Orthopaedics and Trauma Surgery, Musculoskeletal University Center Munich, Ludwig-Maximilians Universität Munich, Marchioninistr. 15, 81377 Munich, Germany; 2 Department of Obstetrics and Prenatal Medicine, Asklepios Klinik Barmbek, Hamburg, Germany; 3 Frankfurt Center of Bone Health and Endocrinology and Orthopedic Center for Musculoskeletal Research, University of Wuerzburg, Würzburg, Germany; 4 Frankfurt Center of Bone Health and Endocrinology and Phillipps-University of Marburg, Marburg, Germany

**Keywords:** Osteoporosis, Adherence, Oral bisphosphonates, Patient reminder program

## Abstract

Poor adherence to oral bisphosphonate therapy remains a major challenge in the treatment of osteoporosis, substantially reducing therapeutic efficacy. While reminder interventions have been proposed as a method to enhance adherence, evidence remains limited. This study aimed to evaluate the impact of written and verbal reminders on medication adherence compared to standard patient care over a 12-month period in a real-world clinical setting. In this randomized controlled study, 180 postmenopausal women diagnosed with osteoporosis were assigned to one of three groups: standard care (control), written reminder, or verbal reminder. Interventions were administered at five standardized time points. Adherence was defined as intake of ≥80% of prescribed weekly doses (≥42 out of 52 doses) and a ≥35% reduction in serum C-terminal telopeptide of type I collagen (CTX) levels from baseline to 12 months. No significant differences in adherence rates were observed between groups: 53.2% in the control group, 52.0% in the written reminder group, and 52.7% in the verbal reminder group (*χ*^2^ = 0.014; *p* = 0.993). Changes in bone mineral density and serum CTX levels were also comparable across groups. The implementation of standardized written or verbal reminder strategy did not result in a statistically significant improvement in adherence to oral bisphosphonate therapy. Further studies are needed to investigate the reasons for low adherence to treatment.

## Introduction

Osteoporosis is one of the most common diseases in postmenopausal women. In Germany, it is estimated to affect around 6–7 million patients [1]. Due to the high number of fractures that can occur due to osteoporosis, it has a significant impact on quality of life [2]. An increasing number of treatments have been introduced in recent years aimed at inhibiting bone breakdown or stimulating bone formation, including oral and intravenous bisphosphonates, teriparatide, abaloparatide, raloxifene, denosumab, and romosozumab. Yet, the number of patients receiving these effective treatments has remained unchanged or even declined [3]. One of the major contributors to this unfavorable trend is the low patient adherence to treatment [1]. The present study focuses on oral bisphosphonates, which are the most commonly used osteoporosis medication having a favorable cost–benefit ratio. While randomized controlled trials (RCT) have reported high adherence rates over the course of 3–4 years of treatment, real-world evidence studies indicate adherence rates as low as 30% for bisphosphonate therapy after one year of treatment [1, 4, 5]. In instances where patients fail to adhere to the prescribed medication regimen, healthcare professionals often use the term ‘non-compliance’ to describe this behavior. The terms ‘adherence’ and ‘compliance’ are frequently used interchangeably by medical professionals, however it is important to note that these terms are not synonymous [6, 7]. It is therefore of the utmost importance to use the accurate definition when reporting clinical outcomes. The WHO definition from 2003 defines adherence as “the extent to which a person’s behavior—taking medication, following a diet, and/or executing lifestyle changes, corresponds with agreed recommendations from a healthcare provider” emphasizing the agreement between patient and medical practitioner [8]. In contrast, adherence is defined as a person’s autonomy in terms of the facilitation of behavioral modification, while compliance is contingent on treatment irrespective of the absence of behavioral modification [6–9]. Furthermore, adherence is characterized as an active process in which a patient assumes responsibility for their overall wellbeing. In contrast, compliance is a passive behavior in which a patient follows a set of recommendations from a medical practitioner [6–9].

Patients’ knowledge, motivation, and understanding of their disease are critical, as osteoporosis is often asymptomatic, requiring regular reminders and education. Recent studies have emphasized the critical role of adherence in bisphosphonate effectiveness [10]. Caro et al. demonstrated that real-world efficacy decreases by up to 16% at 2 years compared to clinical trials that reported a fracture risk reduction of 50% or more [2]. Furthermore, several studies indicate that only patients with high adherence of 70–80% achieve clinical efficacy comparable to that seen in clinical trials [2]. Although low adherence has long been recognized as a significant unmet medical need, studies examining interventions to improve treatment adherence remain limited. This study aims to evaluate whether a written or verbal patient information program during a one-year oral bisphosphonate therapy improves adherence compared to standard medical care. The present prospective study was designed in accordance with the recommendations of the World Health Organization (WHO), which stipulate that no single measurement strategy has been deemed optimal. The prevailing state-of-the-art in the measurement of adherence behavior entails a multi-method approach that integrates feasible self-reporting with reasonable objective measures [8]

## Methods

### Study Design

This single-center study was designed as a prospective, randomized, three-arm, parallel-group comparison study over a 12-month period. The cohort consisted of 180 postmenopausal women diagnosed with osteoporosis requiring treatment in accordance with local German guidelines [11]. All participants provided written informed consent prior to study enrollment. Participants were required to demonstrate understanding of the study procedures and reminder content during the informed consent process; individuals with cognitive impairments (e.g., dementia) were excluded to ensure adequate comprehension. The study was conducted in accordance with the Declaration of Helsinki and was approved by the local ethics committee. All patients received risedronate 35 mg once a week for at least 12 months, with 60 participants allocated to one of three intervention groups, as defined below. The recruitment process involved extending invitations to participate to all new patients with an indication for an oral bisphosphonate treatment during osteoporosis consultations. Information on previous fragility fractures was collected at baseline using a standardized questionnaire. Vertebral fractures were additionally classified by severity.

### Primary and Secondary Endpoints

The primary endpoint of this study was defined as the number of patients considered to be adherent to their medication in Groups 2 and 3 (intervention groups) combined versus the number patients adherent to their medication in Group 1 (no intervention, control group) after 12 months of treatment.

Secondary endpoints were number of adherent patients in Group 2 versus number of adherent patients in Group 1, number of adherent patients in Group 3 versus number of adherent patients in Group 1, patient self-reported adherence, reasons for non-adherence, and patient satisfaction with the reminder system. In addition, the percentage reduction from baseline in the following clinical parameters of osteoporosis were assessed: bone markers (osteocalcin, bone specific alkaline phosphatase [AP]), serum beta-isomerized C-terminal cross-linked telopeptides of collagen I (CTX), and procollagen type N-terminal propeptide (PINP) in % vs. baseline; quantitative ultra sound (QUS) at os calcaneus (speed of sound [SOS], broadband ultrasound attenuation [BUA], and stiffness index [SI]); bone mineral density (BMD) using dual x-ray absorptiometry (DXA).

### Definition of Intervention

Patients in Group 1 did not receive any treatment reminder. As such, this group was used as the control group. Patients in Group 2 received a standardized written reminder (information letter and information brochures), while a verbal reminder (telephone call) at the same time points (weeks 1, 2, 10, 20, and 33) was used for Group 3. The telephone intervention was carried out by a single trained study nurse using a standardized procedure based on a written template. The aims of the telephone intervention were to remind patients to take their medication, to provide information (side effects, medication), to discuss motivation (importance of the disease), to offer help with practical problems, and to arrange contact with a doctor, if necessary.

The written intervention was carried out as follows: patients received personalized letters with different information brochures. The aims of the written intervention were to remind patients to take their medication, to provide information (side effects, medication), to discuss motivation (importance of the disease), and included the possibility of support via a telephone helpline.

### Definition of Adherence to Medication

Comprehensive adherence assessment utilized a multifaceted approach, incorporating patient self-report, prescription control and bone marker assessment. Interventions were strategically scheduled at weeks 1, 2, 10, 20, and 33. For the measurement of the primary endpoint, patients were classified as ‘adherent to medication’ or ‘non-adherent’ according to the following criteria:

(1) Patient self-report was considered positive if it was reported that 80% or more of the medication had been taken.

(2) Prescription control was considered positive if at least 80% of the required medication had been ordered.

(3) Bone markers were considered positive if serum CTX was 35% below the normal range on the follow-up visit at month 12.

All three parameters had to be positive after 12 months to be classified as"adherent to medication.”

### Measurement of Adherence to Medication

Adherence was assessed in three ways: Patient self-report (questionnaire), prescription review, and bone marker changes.

Patient self-report questionnaire: In a questionnaire, patients were asked to rate themselves as adherent or non-adherent to intake of medication. They were also asked about their regular use of medication and possible reasons for not taking the medication (e.g., forgot to take the medication). Other items included possible side effects and possible discontinuation of medication due to side effects.

Prescription review: The prescriptions that were issued were checked by the clinicians involved in the study. An adherence rate was calculated based on the number of prescriptions issued. However, the issue of a prescription does not necessarily imply that the medication has actually been taken, therefore further calculations based on prescription checks were included in the adherence rate. Adherence was calculated from the prescription check in the following manner: Firstly, duration of therapy in terms of weeks of treatment (WT) was defined as a relevant factor for a better assessment of adherence. Two different options were calculated:

(a) Individual duration of therapy: Period between the date of last visit and the randomization date (if there was no last visit, the last prescription date was used; this applied to seven cases). If the period between the last visit and the randomization date was less than 42 weeks, the treatment duration was automatically set to 42 weeks.

(b) Static treatment duration: 52 weeks. If an initial statistical evaluation (Chi-squared test) showed no differences between the intervention groups, adherence to a static treatment duration of 52 weeks was defined as adherence by prescription control for all subsequent evaluations.

### Changes in Bone Markers

Two markers in particular were examined over time with regard to bone marker changes: CrossLaps (CTX) and P1NP were evaluated after an overnight fasting and blood was withdrawn in the morning. The change between baseline measurement (before the start of therapy) and ideally 12 months after the start of therapy was calculated. If the reduction in CrossLaps was greater than 35%, the patient was considered as taking the medication and therefore being adherent to treatment. Although additional bone turnover markers such as P1NP were assessed, only CrossLaps (CTX) values were included in the analysis in accordance with the predefined adherence criteria.

### Statistical Analysis

Anticipated medication adherence improvement in this setting ranges from 10 to 57% based on previous studies [12, 13]. Assuming a baseline adherence rate of 50% in Group 1 (no intervention) after one year, a substantial increase of 30–80% in patients adherent to medication for intervention groups (Group 2 and 3) is considered meaningful. Employing a significance level of *α* = 2.5% (two sided) and adjusting for multiple comparisons, we aimed to detect a cumulative adherence increase of 30%. To achieve a statistical power of 90.64% in the primary analysis, 52 patients per arm were required for pooled data from both intervention groups (Group 2 and 3). Factoring in a 10% dropout rate and with three groups, we calculated a requirement of 58 patients per group using the Fisher exact *t*-test. Rounding up, a total of 60 patients per group was determined, leading to the inclusion of 180 patients in the overall study.

## Results

Postmenopausal women with osteoporosis were randomized to one of the 3 groups (1:1:1) and there were no significant differences were observed between the three groups with regard to baseline parameters such as age, height, weight, body mass index (BMI), number of births, age at menarche, age at menopause, or prevalent fractures.

Mean ± standard deviation (SD) age was 69.1 ± 8.8 years in the control group, 69.2 ± 8.2 years in the verbal information group, and 67.6 ± 10.7 years in the written information group. All patients were categorized as Caucasian. A total of 180 patients were found to be eligible for participation in the study, but two did not meet one of the inclusion criteria (1 had early dementia, 1 had externally administered medication), leaving 178 patients (59 in the control group [33.1%], 58 in the verbal information group [32.6%], and 61 in the written information group [34.3%]) who were enrolled. Information on prior fragility fractures was collected at baseline via questionnaire. The proportion of participants reporting previous fractures was similar across all study groups (control group: 56.9%, written reminder group: 61.4%, oral reminder group: 58.6%), with no statistically significant differences observed between them. Vertebral fractures were further assessed and classified by severity, again showing no significant group differences.

### Results: Self-Reported Adherence to Medication

A self-report questionnaire was used to assess patients’ adherence (frequency of use) and possible reasons for not taking the medication. The questionnaire asked how often the medication was taken or forgotten. Five different options were possible: never; not often, only when reminded; sometimes forgotten; initially yes, then less often; always. Patients in the control group rated themselves as more adherent to medication than those in the two intervention groups. Patients in the written intervention group rated themselves as the least adherent. Overall, however, the vast majority rater themselves as adherent to medication in the self-report questionnaire (Table 1). The questionnaire also enquired whether the medication had been discontinued due to the occurrence of potential adverse events. The following adverse events were reported: obstipation, diarrhea, abdominal pain, heartburn, nausea, bone and muscle pain, and ‘other.’ In this study, the self-reported occurrence of adverse events differed between the control group and the intervention groups. In the control group, the most prevalent adverse events were reported as ‘other’ side effects (33%), followed by heartburn (22.2%) and bone and muscle pain (22.2%). In contrast, in the verbal intervention groups, abdominal pain was the most frequently reported adverse event (40%), and the same was observed in the written intervention group (27.5%). The patient questionnaire included a self-report part of medication frequency. This showed the highest estimate of regular medication use in the control group at 96.3%, followed by the verbal intervention group at 94.3%, and the lowest in the written intervention group at 85.5%. In addition, the frequency of use was asked in more detail in order to identify possible patterns. Here the most common response in the verbal group was ‘sometimes forgotten’ (5.7%) and in the written intervention group ‘initially yes, then less often’ (7.3%) (Table 2), with no significant differences between the groups.

**Table 1:** Self-reported adherence to medication

	Control		Verbal		Written		Total	
	*N*	%	*N*	%	*N*	%	*N*	%
Adherent	49	90.7	49	89.1	48	87.3	146	89.0
Non- adherent	5	9.3	6	10.9	7	12.7	18	11.0
Total	54	100.0	55	100.0	55	100.0	164	100.0

**Table 2:** Medication use has been…. [%]

	Control		Verbal		Written		Total	
	*N*	%	*N*	%	*N*	%	*N*	%
Never	0	0.0	0	0.0	1	1.8	1	0.6
Not often, only when reminded	1	1.9	0	0.0	2	3.6	3	1.9
Sometimes forgotten	0	0.0	3	5.7	1	1.8	4	2.5
Initially yes, then less often	1	1.9	0	0.0	4	7.3	5	3.1
Always	52	96.3	50	94.3	47	85.5	149	92.0
Total	54	100.0	53	100.0	55	100.0	162	100.0

### Results: Prescription Monitoring

There was no statistical difference between adherence calculated from the number of medications prescribed during the treatment period (set to 52 weeks) or adherence calculated individually (based on the actual duration of treatment) (Chi-squared test: *χ*^2^ = 0.49; *p* = 0.784) or (Chi-squared test: *χ*^2^ = 0.35; *p* = 0.841). Therefore, for all subsequent analyses, adherence to a static treatment duration of 52 weeks was defined as adherence by prescription check. The Chi-squared test showed no statistically significant differences in the distribution of adherence after prescription check between the three intervention groups.

A total of 708 prescriptions were documented. The mean ± SD number of prescriptions per patient was 4.1 ± 1.6; minimum 1.0; maximum 12.0). The majority of prescriptions (72.3%; *n* = 512) were issued by the osteoporosis treatment center, while 27.7% (*n* = 196) were issued by external physicians (Chi-squared test: *χ*^2^ = 0.68; *p* = 0.771). In 61.2% (*n* = 120) the medication was prescribed by a general practitioner and in 35.8% (*n* = 76) by ‘other’ physicians (Chi-squared test: *χ*^2^ = 0.16; *p* = 0.683).

The mean ± SD duration of treatment for the whole population (*n* = 164) was 53.9 ± 5.7 weeks. Duration of treatment was not normally distributed. The Kruskal–Wallis one-way analysis of variance did not indicate significant group differences. There was no correlation between final adherence and duration of treatment according to Spearman-Rho test (*r* = −0.017; *p* = 0.833).

The medication possession ratio (MPR) was calculated as:

ATM = number of days with medication supplied; A_1_L = number of days between first and last medication dispensing; A_M_L = number of days with medication supplied after last medication dispensing.

Results for the MPR item were not normally distributed. The Kruskal–Wallis one-way analysis of variance provided no indication of significant group differences (Table 3). The correlation between final adherence and MPR was highly significant (Spearman-Rho test; *r* = 0.62; *p* ≤ 0.001).

**Table 3:** Medication possession ratio (MPR)

Control			Verbal			Written			Total		
*N*	Mean	SD	*N*	Mean	SD	*N*	Mean	SD	*N*	Mean	SD
53	87.4	37.9	59	86.6	36.4	56	86.3	35.3	168	86.8	36.3

### Results: Evaluation of Changes in Bone Markers

A total of 160 patients provided a second blood sample at month 12 that could be used to assess the additional adherence requirement (a decrease in serum CrossLaps (CX) of >35%).

The non-parametric Wilcoxon test was used for paired samples as the bone marker CrossLaps were not sufficiently normally distributed. For patient's non-adherent to medication, there was no significant change overall (*Z* = 1.79; *p* = 0.07) for any of the intervention groups. In contrast, in patients classified as adherent to medication based on a bone marker decrease of at least 35% (CrossLaps), the decrease in CrossLaps from 0.42 to 0.15 ng/ml was significant (*Z* = 20.44; *p* ≤ 0.001). A differentiation by intervention group indicates a significant decrease for the control group (*Z* = 5.23; *p* ≤ 0.001), for the verbal (*Z* = 5.65; *p* ≤ 0.001), and for the written intervention group (*Z* = 5.09; *p* ≤ 0.001). However, when adherence was categorized based on prescription control, as presented in Table 4, The bone marker CrossLaps is not sufficiently normally distributed, so the non-parametric Wilcoxon signed rank test was used for paired samples in non-adherent patients, an overall non-significant decrease in bone resorption markers CrossLaps (CTX) was observed (*Z* = 1.79; *p* = 0.073). When assessed for medication-adherent patients according to intervention group, there was a significant decrease for controls (*Z* = 2.38; *p* = 0.017), verbal (*Z* = 3.51; *p* ≤ 0.001), and written intervention groups (*Z* = 2.74; *p* = 0.006).

**Table 4:** Adherence to medication interpreted in accordance with the serum CTX bone marker

	Control group		Verbal reminder		Written reminder		Total number	
	*N*	%	*N*	%	*N*	%	*N*	%
Non-adherent	16	30.8	15	26.3	17	33.3	48	30.0
Adherent	36	69.2	42	73.7	34	66.7	112	70.0
Total number	52	100.0	57	100.0	51	100.0	160	100.0

The Chi-squared test shows no significant differences in the distribution between the intervention groups (*χ*^2^ = 1.07; *p* = 0.601)

In an exploratory subgroup analysis of patients classified as adherent to medication based solely on prescription control comprised a significant reduction in CrossLaps (serum CTX), which fell from 0.39 to 0.17 ng/ml (*Z* = 8.4; *p* ≤ 0.001). When differentiated by intervention group, there was a significant decrease for controls (*Z* = 4.75; *p* ≤ 0.001), verbal (*Z* = 4.89; *p* ≤ 0.001), and written intervention groups (*Z* = 4.89; *p* ≤ 0.001) (Table 4).

In conclusion, the verbal intervention contributed in particular to a significant reduction of the bone marker CrossLaps, which is an indirect indicator of improved medication adherence.

### Results: Calculation of Adherence to Medication

Out of 152 patients, final adherence was calculated by defining as adherent those patients, who were classified as adherent to all three parameters according to the given definitions. The rate of patients classified as ‘adherent’ to all three parameters (>80% medication from questionnaire, prescription control, and >35% CTX reduction) did not differ significantly between the individual intervention groups and the control group: there was a adherence rate of 53.2% in the control group, 52.7% in the verbal intervention group (telephone call), and 52% in the written intervention group (Table 5).

**Table 5:** Adherence final (patient self-report, prescription check, bone markers)

	Control group		Verbal reminder		Written reminder		Total number	
	*N*	(%)	*N*	(%)	*N*	(%)	*N*	(%)
Adherent	25	53.2	29	52.7	26	52.0	80	52.6
Non-adherent	22	46.8	26	47.3	24	48.0	72	47.4
Total number	47	100.0	55	100.0	50	100.0	152	100.0

**Fig. 1:**
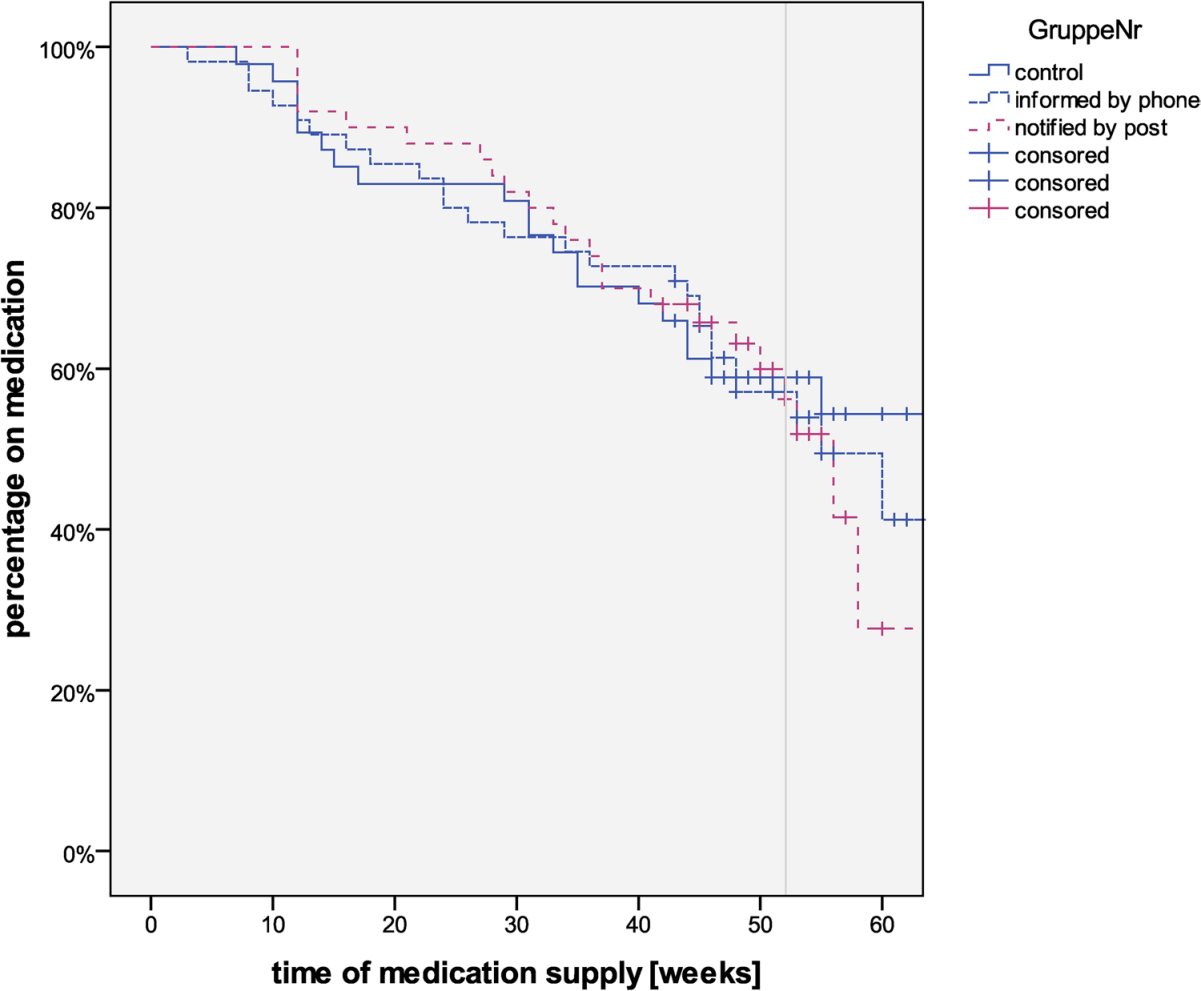
Kaplan–Meier Curve for ‘total adherence’ (out of patient self-report, recipient-control, and bone marker) grouped by type of intervention

The evaluation of the secondary endpoints was carried out with regard to adherence, according to intervention group and assessment method. There were no significant differences between the control group and the verbal and written intervention groups in terms of adherence to medication by assessment method. For the control group, 90.7% of patients were considered adherent based on the questionnaire, 69.2% based on the reduction of bone markers, and 63.6% based on the prescription check. For the verbal intervention group, adherence was 89.1% based on the questionnaire, 73.7% based on bone marker reduction, and 69.5% based on the prescription check. In the written intervention group, adherence was 87.3% based on the questionnaire, 66.7% based on the reduction in bone markers, and 64.9% based on the prescription check (Fig. 1).

## Discussion

Non-adherence to bisphosphonates increases the risk of fracture due to inadequate suppression of bone resorption. Patients who stop treatment or do not adhere to their prescribed regimen are at increased risk of osteoporotic fractures, leading to significant morbidity and mortality [13, 15].

Inadequate treatment may also result in a reduction in BMD over time, undermining the benefits of the medication and potentially accelerating disease progression [14]. Additionally, the financial burden of managing fractures, including hospitalization, surgery, and long-term care, further underscores the importance of adherence to osteoporosis therapy [15].

Bisphosphonates are among the most commonly prescribed medications for the treatment of osteoporosis [1]. They are widely used due to their efficacy, cost-effectiveness, and convenient dosing schedule compared to intravenous formulations [16]. However, patient adherence with oral bisphosphonates is a significant challenge that impacts the effectiveness of osteoporosis treatment [17, 18]. In our study, adherence to weekly bisphosphonate therapy was higher than expected [19].

The present study found no significant effect of written (letter) or verbal (telephone call) interventions on patient adherence compared to regular patient care. Interestingly, the control group rated their adherence higher than the intervention groups, with the written intervention group reporting the lowest adherence. Between 85 and 96% of patients claimed to have ‘always’ taken their medication, with the control group almost 100% convinced. The notably high self-reported adherence rates (>85% across all groups) contrast with the objectively measured final adherence of approximately 52%, which may indicate the presence of self-report bias. This discrepancy, especially the paradoxical finding that patients in the control group reported higher adherence than those receiving reminders, suggests that social desirability or recall bias could have influenced the self-reported data. While self-report measures were included as part of our assessment, we acknowledge their limitations and therefore focus our interpretation primarily on more objective adherence parameters collected in the study. These objective measures provide a more reliable basis for evaluating adherence behavior. Participants were not required to return or present remaining medication, as this method was considered potentially unreliable.

Despite interventions, medication adherence appeared to decrease. This might be explained be the fact that the written intervention, despite being personally addressed, included general information leaflets that might not have been read properly or may have induced a sense of guilt, leading to lower self-rated adherence. All groups, however, rated themselves higher than the primary endpoint, possibly because patients didn’t perceive osteoporosis as an urgent risk.

The literature on studies that have used reminder systems to improve adherence to oral bisphosphonates is limited. In one study in veterans, weekly text reminders improved adherence to oral bisphosphonate therapy. The main reason for non-adherence in this study was ‘missed doses’ [20]. Another study group was able to improve adherence by intervening earlier with an interactive voice response system, followed by a written intervention. This form of early and combined reminder achieved a significant improvement over the control group. However, the group sizes are smaller than in our study [21].

Adjusting the frequency and route of administration is further option to improve adherence to oral bisphosphonates and osteoporosis-specific therapies. A retrospective study showed that adherence with weekly dosing of osteoporosis drugs was lower than with less frequent dosing. Adherence was higher with 3- or 6-month dosing schedules (e.g., intravenous ibandronate every 3 months, subcutaneous denosumab every 6 months) than with oral weekly dosing. Daily or monthly dosing was associated with a higher risk of non-adherence than weekly dosing [19].

Nevertheless, the route of administration does not seem to be a decisive factor. Osteoporosis is often asymptomatic until a fracture occurs, and patients may not perceive an immediate benefit from the treatment, which can reduce motivation for adherence. The absence of obvious symptoms can lead to the misconception that the disease is either non-progressive or easily manageable without medication. This psychological barrier can significantly contribute to non-adherence.

Recent studies have investigated novel routes of administration beyond intravenous or oral administration. In particular, studies using transdermal application have been introduced [22, 23]. In a recent proof-of-concept study in ovariectomized Sprague–Dawley rats, Ripolin et al. achieved interesting results investigating a transdermal form of bisphosphonates [24]. This approach could improve adherence by avoiding gastrointestinal side effects, as the transdermal route may be better tolerated.

There is still uncertainty about the evaluation of prescriptions rates, as a prescription does not necessarily imply that the medication was actually taken. Therefore, studies on prescription control need to be performed in the context of the other adherence criteria before drawing any firm conclusions [8, 15, 19]. In the current study, adherence was independent of the location of prescription, i.e., neither the osteoporosis center nor at the general practitioner had a relevant influence on patient adherence.

The use the bone markers CroosLapsap (and P1NP) is a useful short-term tool to monitor the effectiveness of oral bisphosphonate therapy[25, 26]. The reduction of this bone marker showed clear and significant differences between patients’ adherent to medication and those not being adherent. In non-adherent patients, a non-significant decrease in CrossLaps (serum CTX was observed. This effect may be attributed to partial medication intake, as these individuals were not fully non-adherent but likely took risedronate irregularly or at a lower frequency. The biochemical response thus suggests limited treatment efficacy in this group. These findings underscore the clinical relevance of consistent and adequate adherence to ensure optimal therapeutic outcomes in osteoporosis management.

There were no significant differences in the reduction of bone markers between patients defined as adherent to medication in the intervention groups. This could be a practical option to improve the patients’ motivation: a regular consultation with the physician and a review of current bone marker levels to improve adherence [18, 25, 26].

Although data on previous fragility fractures were collected at baseline, no information was available regarding the time interval between fracture and study enrollment. Moreover, no subgroup analyses were conducted to examine potential differences in adherence between participants with and without a history of fractures. Future studies should explore whether fracture history and timing may influence adherence, as subgroup analyses in this regard could yield important insights.

Potential strategies to improve adherence may include a simplification of the dosing regimen, reducing administration frequency, such as switching from weekly to monthly doses, may improve adherence. Newer formulations like alternative administration routes (e.g., transdermal) can also alleviate adherence burdens, especially in patients experiencing gastrointestinal side effects with oral medications. In addition, patient education and engagement may be achieved by providing accessible information about osteoporosis and the role of bisphosphonates in fracture prevention which may enhance patient understanding and adherence. Educational interventions highlighting long-term benefits, along with reminders and follow-up, can motivate continued treatment. Emphasizing the asymptomatic nature of osteoporosis is essential for promoting preventive care.

Management of side effects, especially addressing gastrointestinal side effects can improve adherence. For patients with intolerable symptoms, alternatives like denosumab or selective estrogen receptor modulators (SERMs) could be considered. The use of reminder systems by technological tools, such as smartphone apps or automated reminders, along with family support and regular follow-up, can encourage consistent medication adherence. Regular monitoring and feedback by periodic assessments of BMD and bone turnover markers can reinforce treatment benefits. Discussing adherence challenges during follow-up appointments offers opportunities for treatment adjustments if needed.

In conclusion oral bisphosphonates effectively reduce fracture risk and remain central to osteoporosis treatment. However, suboptimal adherence to treatment remains a significant challenge impacting clinical outcomes. In our study, written or verbal reminder systems did not significantly enhance adherence. As an outlook for future research, it may be worth exploring whether personal contact—such as in-person consultations in outpatient settings and direct communication—could serve as effective strategies to improve adherence. Additionally, improving patient education, simplifying therapy, and increasing awareness of potential side effects and their management are important considerations. We also anticipate that the use of digital reminders in the coming years may further engage patients and improve adherence. Ultimately, the reduction in fracture risk associated with bisphosphonates relies on adherence, which is crucial for preventing fractures and enhancing the quality of life for patients with osteoporosis. 
